# Genetic mapping of deoxynivalenol and fusarium damaged kernel resistance in an adapted durum wheat population

**DOI:** 10.1186/s12870-023-04708-8

**Published:** 2024-03-12

**Authors:** Samia Berraies, Yuefeng Ruan, Ron Knox, Ron DePauw, Firdissa Bokore, Richard Cuthbert, Barbara Blackwell, Maria Antonia Henriquez, David Konkin, Bianyun Yu, Curtis Pozniak, Brad Meyer

**Affiliations:** 1 Swift Current Research and Development Center, Agriculture and Agri-Food Canada, Swift Current, SK S9H 3X2 Canada; 2 Agriculture and Agri-Food Canada (Retired), Ottawa, Canada; 3 Present Address: Advancing Wheat Technologies, Calgary, AB T3H 1P3 Canada; 4 Ottawa Research and Development Centre, Agriculture and Agri-Food Canada, Ottawa, ON K1A 0C6 Canada; 5 Morden Research and Development Centre, Agriculture and Agri-Food Canada, Morden, MB R6M 1Y5 Canada; 6 National Research Council Canada, Aquatic and Crop Resource Development, Saskatoon, SK S7N 0W9 Canada; 7 Crop Development Centre, Department of Plant Science, University of Saskatchewan, Saskatoon, SK S7N 5A8 Canada

**Keywords:** *Triticum durum*, Fusarium head blight, Deoxynivalenol, Fusarium damaged kernels, Quantitative trait loci, Resistance breeding

## Abstract

**Background:**

Fusarium head blight (FHB) infection results in Fusarium damaged kernels (FDK) and deoxynivalenol (DON) contamination that are downgrading factors at the Canadian elevators. Durum wheat (*Triticum turgidum* L. var. *durum* Desf.) is particularly susceptible to FHB and most of the adapted Canadian durum wheat cultivars are susceptible to moderately susceptible to this disease. However, the durum line DT696 is less susceptible to FHB than commercially grown cultivars. Little is known about genetic variation for durum wheat ability to resist FDK infection and DON accumulation. This study was undertaken to map genetic loci conferring resistance to DON and FDK resistance using a SNP high-density genetic map of a DT707/DT696 DH population and to identify SNP markers useful in marker-assisted breeding. One hundred twenty lines were grown in corn spawn inoculated nurseries near Morden, MB in 2015, 2016 and 2017 and the harvested seeds were evaluated for DON. The genetic map of the population was used in quantitative trait locus analysis performed with MapQTL.6® software.

**Results:**

Four DON accumulation resistance QTL detected in two of the three years were identified on chromosomes 1 A, 5 A (2 loci) and 7 A and two FDK resistance QTL were identified on chromosomes 5 and 7 A in single environments. Although not declared significant due to marginal LOD values, the QTL for FDK on the 5 and 7 A were showing in other years suggesting their effects were real. DT696 contributed the favourable alleles for low DON and FDK on all the chromosomes. Although no resistance loci contributed by DT707, transgressive segregant lines were identified resulting in greater resistance than DT696. Breeder-friendly KASP markers were developed for two of the DON and FDK QTL detected on chromosomes 5 and 7 A. Markers flanking each QTL were physically mapped against the durum wheat reference sequence and candidate genes which might be involved in FDK and DON resistance were identified within the QTL intervals.

**Conclusions:**

The DH lines harboring the desired resistance QTL will serve as useful resources in breeding for FDK and DON resistance in durum wheat. Furthermore, breeder-friendly KASP markers developed during this study will be useful for the selection of durum wheat varieties with low FDK and DON levels in durum wheat breeding programs.

**Supplementary Information:**

The online version contains supplementary material available at 10.1186/s12870-023-04708-8.

## Background

Commercial varieties of durum wheat [*Triticum turgidum L. subsp. durum* (*Desf.*)] are currently susceptible to moderately susceptible to Fusarium head blight (FHB) caused by *Fusarium graminearum* (teleomorph *Gibberella zeae* Schwabe), a disease that may cause severe losses of grain yield and quality thus downgrading the grain quality [1]. Canada is a major producer of durum wheat and accounts for about half of the world’s total exported durum [2]. Canada Western Amber Durum (CWAD) wheat is a premium market-class grown on over two million hectares in Western Canada. The high quality that Canadian durum wheat offers is a distinction in international markets making paramount the maintenance of the high standard of crop cleanliness, consistency and food safety. FHB has grown to be one of the biggest challenges in CWAD production. Several moderate to severe FHB epidemics on the Canadian Prairies resulted in serious economic impact on the durum wheat industry in the last two decades [3].

FHB leads to significant yield losses due to shrivelled kernels [4]. However, the major concern is the contamination of the crop with deoxynivalenol (DON), the main FHB associated mycotoxin. The most recent FHB epidemic, for example, occurred in Saskatchewan during which Canada had 84% of its harvested grain samples with Fusarium damaged kernels (FDK) that resulted in downgrading the grain quality and an estimated economic loss of $1 billion [5]. The presence of DON in infected grain further exacerbates the economic losses caused by FHB [6]. Durum wheat is mostly used for human consumption, and the risk of toxin-contaminated grain entering the food chain is consequently particularly high [7]. The exposure to DON may cause serious health risk, the reason why Health Canada regulations set the maximum allowed levels at 1 to 2 ppm in food products depending on the use.

The presence of DON in FHB contaminated grains has been reported to have a positive linear relationship with FHB visual symptoms (incidence and severity) and with FDK, indeed the higher the incidence, the severity, and the FDK the higher the level of mycotoxin [8]. However, other studies reported that direct prediction of DON contamination cannot be based on FHB disease symptoms such as crop yield loss, or FDK [9]. Even though most studies reported positive correlations between disease symptoms and mycotoxin accumulation, the quantity of mycotoxins per unit of disease index differed considerably and some studies have not reported significant relationships [10]. The quantity of DON accumulated depends on the host and fungal genotypes as well as environmental conditions [8] making it a complex trait for intervention.

As conventional agrochemical practices are costly and only partially effective, the development and deployment of germplasm with improved FHB and DON resistance is a complementary control strategy that is environmentally sustainable as part of integrated disease management. In hexaploid wheat various sources of resistance have been identified, genetically characterized, and successfully utilized in developing FHB and DON resistant cultivars [11, 12]. However, most current durum wheat cultivars are highly susceptible to FHB and DON accumulation because of the very limited sources of effective FHB and DON resistance in durum wheat available for breeding and difficulties in efficiently combining the numerous small-effect resistance genes in durum germplasm [13–17]. Durum wheat breeding for FHB and DON resistance is very challenging due to the dearth of resistance sources in the tetraploid gene pool [18]. Compared to common wheat, some studies revealed that finding resistance to FHB in durum wheat is challenging [18, 19], but some cultivated subspecies of *T. turgidum* such as *ssp. Carthlicum* and *ssp. dicoccum* genotypes can be exploited due to a moderate resistance which limits the loss of production and accumulation of mycotoxins. Despite these challenges, durum wheat cultivars with an improved level of resistance have been developed by the accumulation of native minor genes for FHB resistance [17]. Cultivars with improved levels within a moderately susceptible category have been released in Canada such as Brigade [20], Transcend [21] and in North Dakota using a similar approach [19]. In 2021, the cultivar AAC Shrader was the first durum wheat to be assigned an intermediate level of resistance (Ruan et al., unpublished).

Resistance to DON accumulation has been demonstrated to play a major role in limiting progress of *F. graminearum* in wheat [22]. Although reducing the toxin concentration in grains is a major target for breeders, direct selection for DON resistance on a large scale remains impractical due to its phenotyping costs [23]. Therefore, attempts have been made to relate FHB incidence and/or severity resistance to DON resistance to determine if cultivars could be selected based on disease symptoms to ensure low levels of DON [24]. Numerous studies reported quantitative trait loci (QTL) simultaneously associated with FHB incidence and severity that were also associated with low DON accumulation in tetraploid and hexaploid wheat [15, 24, 25]. However, a few QTL have been tested and validated for possessing factors with the ability to either detoxify DON or enhance resistance to DON accumulation [22]. Other studies demonstrated that resistance to DON accumulation and FHB symptoms could involve different genes [25–27]. In hexaploid wheat, QTL with small or moderate effects exclusively associated with low DON accumulation were mapped on chromosomes 2AS, 2DS, 3 A, 3BL, 3DL, 4B, 5AS, 7 A and 7B [25, 26, 28–32]. Counter to hexaploid wheat, a limited number of minor QTL associated with FHB resistance have been reported in durum wheat [11, 16] and only a few investigations have involved QTL analysis of resistance to DON. Ruan et al. [33] identified two QTL for resistance to the 3-acetyl-deoxynivalenol (3-ADON) chemotype on chromosomes 1B and 4B using a backcross recombinant inbred line (BCRIL) tetraploid wheat population genotyped using DArT and microsatellite markers. A diverse panel of durum germplasm lines were phenotyped for FHB incidence and severity and subsequently genotyped to perform genome wide association analysis [1]. Thirty-one QTL across all 14 chromosomes were significantly associated with FHB resistance. Unfortunately, among the 31 QTL several of them were associated with plant height and/or flowering time. Only six QTL were associated with FHB resistance and not associated or weakly associated with plant height and flowering time. High density genetic mapping of FHB resistance in two tetraploid wheat populations identified five QTL in the DT707/DT696 population and seven QTL in Strongfield/Blackbird population [34]. Although, they reported co-location of some FHB resistance with plant height QTL and/or time to maturity in both populations, several of the QTL were not associated with either plant height or maturity.

With the advent of next-generation sequencing technology, a high quantity of Single Nucleotide Polymorphism (SNP) markers became available allowing high density genotyping and mapping. The Illumina iSelect 90 K wheat array [35] is a rich collection of wheat SNP markers that is being used worldwide, providing great opportunities for high precision QTL mapping and genome-wide association studies. The availability of a high-density consensus map for tetraploid wheat with the SNP markers from the iSelect 90 K wheat array such as Wang et al. [35] and Maccaferri et al. [36] improves cross referencing QTL among studies. The aim of the present work was to characterize and map genetic loci conferring DON and FDK response using the high-density genetic map of the DT707/DT696 DH population and to identify linked SNP markers useful for gene pyramiding and marker-assisted breeding.

## Results

### FDK and DON variation and correlation

**Fig. 1:**
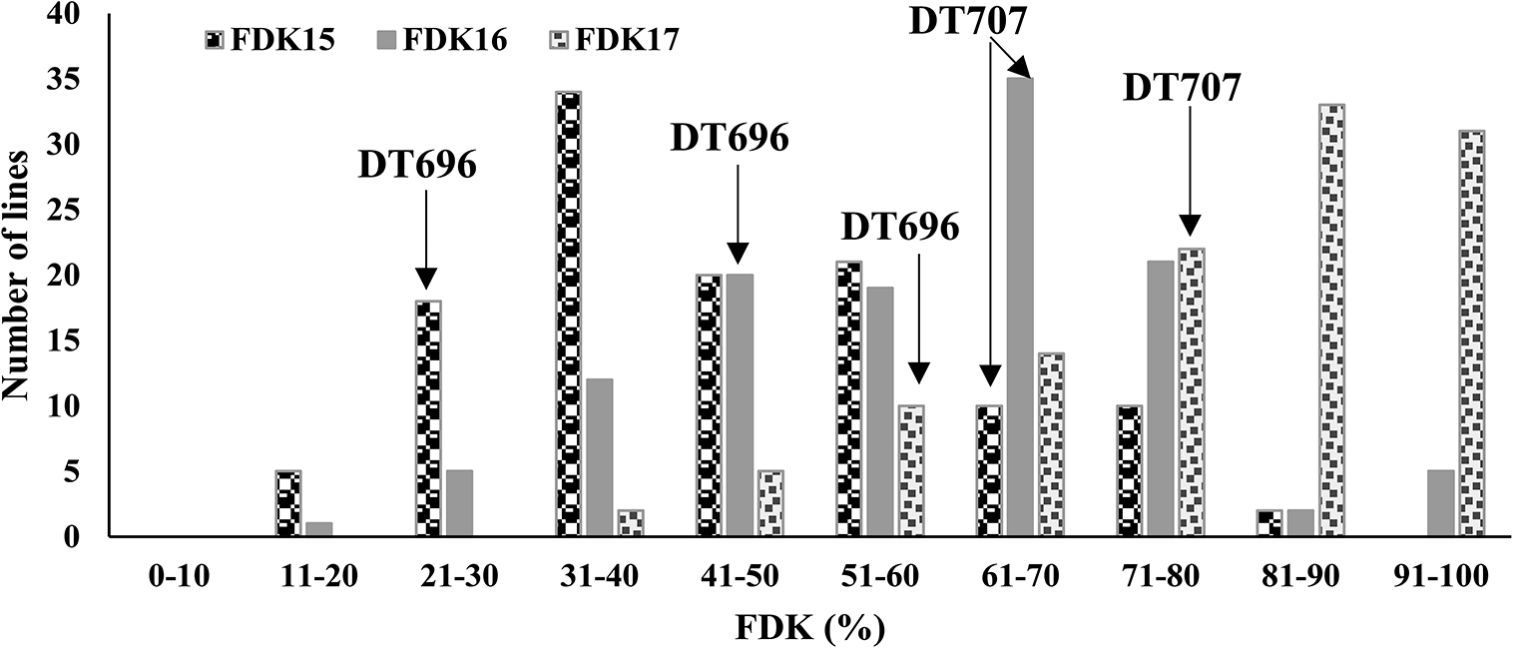
Frequency distribution of 120 DT707/DT696 DH lines grown in 2015, 2016 and 2017 for fusarium damaged kernels (FDK). The placement of the parents along the distribution is denoted by arrows

**Fig. 2:**
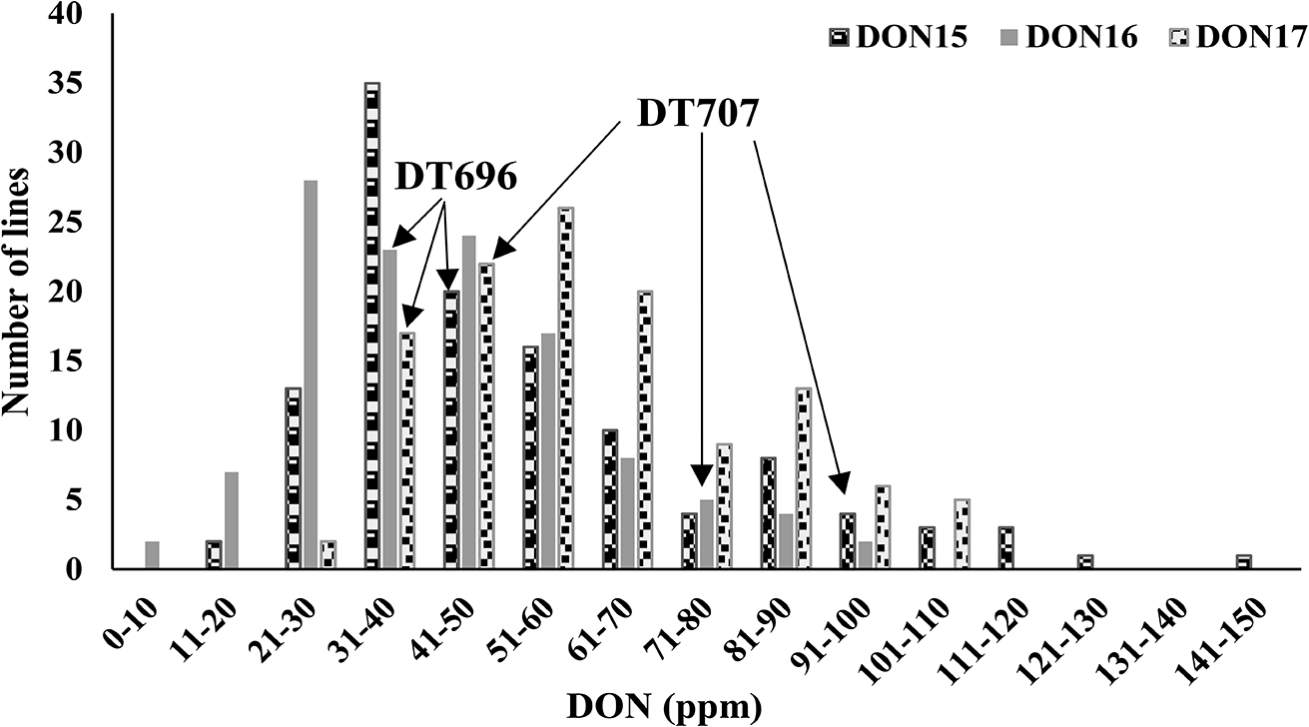
Frequency distribution of 120 DT707/DT696 DH lines grown in 2015, 2016 and 2017 for Deoxynivalenol concentration (DON). The placement of the parents along the distribution is denoted by arrows

The susceptible FHB line DT707 showed, without exception, relatively higher levels of FDK and DON than DT696 over the years (Table 1). The range of FDK values for the population was wider than that of the parents, reflecting the presence of lines that may have segregated from the population with lower FDK rates than DT696 and greater FDK rates than DT707 (Fig. 1). The population mean for both FDK and DON values were generally higher in 2017 than the other two years (Figs. 1 and 2). A wide distribution of FDK (Fig. 1) and DON (Fig. 2) were observed for the 120 DH lines each year. In all years, the distributions for DON content were continuous and similarly shaped being skewed to the right with a preponderance of low DON lines. Out of the 120 DH lines evaluated for DON, lines with consistently low DON were not identified. A very variable DON content across the test years 2015, 2016 and 2017 was noticed, for example being as resistant as DT696 in one year and nearly as susceptible as DT707 in another year.

**Table 1:** Analysis of variance, mean, minimum (Min) and maximum (Max) of Fusarium damaged kernels (FDK) and deoxynivalenol (DON) of parents and DT707/DT696 DH lines (n = 120) at FHB nursery near Morden from 2015 to 2017

Trait	Environment		Parental lines (mean)				Population				
			DT696	DT707	Diff.^a^		Mean	Min-Max	STD^c^ DEV	STD Error	*P* value^b^
FDK (%)	2015		29.7	61.5	***		44.0	10.6–87.9	15.9	1.5	***
	2016		47.0	66.2	ns		58.3	11.9–96.5	17.0	1.6	
	2017		57.3	71.9	ns		78.2	34.6–100	15.4	1.4	
DON (ppm)	2015		40.3	92.6	ns		52.8	16.1-141.8	25.7	2.3	***
	2016		31.4	77.3	**		42.1	8.9–96.7	18.8	1.7	
	2017		39.0	50.0	ns		60.7	25.6-107.8	19.5	1.8	

^a^Difference between means of parents

^b^*P* value of line variance: ns: Non-significant at *P* > 0.05; *P* ≤ 0.05, ** for *P* ≤ 0.01 and *** for *P* ≤ 0.001

^c^STD: standard; DEV: deviation

The correlation coefficients calculated between each trait among years are shown in Table 2. Table S8 of additional file 1 displays the correlation of FDK and DON with FHB incidence and severity. Positive correlations were found between FDK among years, between DON among years, and between FDK and DON among years, but some of them were non-significant. The highest correlation coefficients with the highest level of significance (*P* ≤ 0.001) were observed between FDK and DON concentrations from the same year. FHB incidence and severity were statistically significantly correlated with FDK and DON at Morden in 2015, 2016 and 2017, but low or non-significant correlations were observed with the data from Indian Head and at Swift Current greenhouse data. A slightly high broad-sense heritability value of 44.4% was calculated for the FDK compared to 37.2% for the DON content.

**Table 2:** Spearman’s correlation coefficients between Fusarium damaged kernels (FDK) and deoxynivalenol (DON) measured over yearsb on the 120 lines of the DT696/DT707

	DON15^a^	DON16	DON17	FDK15	FDK16	FDK17
DON15	1					
DON16	0.27**	1				
DON17	ns	ns	1			
FDK15	0.80***	0.25**	0.23*	1		
FDK16	ns	0.65***	ns	0.21*	1	
FDK17	ns	ns	0.70***	0.21*	ns	1

^a^ns: Non-significant for *P* > 0.05, * for *P* ≤ 0.05, ** for *P* ≤ 0.01 and *** for *P* ≤ 0.001

^b^15: 2015; 16: 2016; 17: 2017

### QTL mapping

QTL mapping of FDK and DON for each of the three years using MQM detected four genomic regions responsible for FDK and DON resistance on chromosomes 1 A, 5 A (2 loci) and 7 A (Table 3). The favourable alleles for low DON and FDK on all the chromosomes were derived from DT696, no QTL was detected from DT707. The majority of the QTL were identified in 2015 with the QTL that was detected on chromosome 5 A associated with DON was additionally detected in 2017. QTL for resistance to FDK were mapped on chromosomes 5 A (*Qfdk.spa-5 A.1*) and 7 A (*Qfdk.spa-7 A*) in 2015 accounting together for a total phenotypic variation explained (PVE) at 34.9%. Individually, *Qfdk.spa-5 A.1* accounted for the most PVE for FDK at 23.9%. The analysis identified QTL for DON resistance on chromosomes 1 A (*Qdon.spa-1 A*) and 7 A (*Qdon.spa-7 A*) in 2015 (Table 3). The phenotypic variation explained was lower (9.9%) for *Qdon.spa-1 A* than *Qdon.spa-7 A* (13.3%). Two QTL for reduced DON were also mapped on chromosome 5 A at different positions on the genetic map and were designated as *Qdon.spa-5 A.1* for the QTL identified in 2015 and *Qfhb.spa-5 A.2* for the QTL identified in 2017. The DON QTL co-located with FDK QTL on chromosomes 5 A.1 and 7 A.

**Table 3:** Summary of Fusarium damaged kernels (FDK) and deoxynivalenol (DON) QTL detected from the 120 lines of the DT696 / DT707 population, number of years tested, years when QTL was revealed, trait, absolute position on the genetic map (cM), markers associated with the highest LOD values, LOD score, percentage of phenotypic variation explained (PVE) and resistance allele contributing parent

Chr.^a^	QTL	No. of years tested^b^	Year of QTL	Trait	QTL peak map position (cM)	QTL interval (cM)	Markers with the highest LOD	LOD	PVE (%)	QTL contributor
1 A	*Qdon.spa-1 A*	3	2015	DON	53.21	44.2–58.9	*Excalibur_c2389_361/BS00062869_51*	3.9	9.9	DT696
5 A	*Qdon.spa-5 A.1*	3	2015	DON	14.04	8.1–14.1	*Tdurum_contig5481_252/tplb0039m09_92*	3.6	13.0	DT696
	*Qfdk.spa-5 A.1*	3	2015	FDK	16.19	16.19–16.67	*Tdurum_contig4731_1108*	7.1	23.9	DT696
	*Qdon.spa-5 A.2*	3	2017	DON	5.22	4.5–5.2	*Excalibur_c64265_224*	3.5	12.4	DT696
	*Qdon.spa-5 A.1*	3	over years^c^	DON	16.90	16.90-17.14	*Tdurum_contig88305_380*	4.5	15.7	DT696
	*Qfdk.spa-5 A.1*	3	over years	FDK	17.14	16.90-17.14	*BS00089968_51*	5.2	17.9	DT696
7 A	*Qdon.spa-7 A*	3	2015	DON	79.94	78.9–80.6	*TA006231-0789/Kukri_c19696_60*	3.8	13.3	DT696
	*Qfdk.spa-7 A*	3	2015	FDK	79.94	78.9–80.6	*TA006231-0789/Kukri_c19696_60*	3.1	11.0	DT696

^a^Chr.: chromosome

^b^No.: number

^c^QTL analysis based on BLUP values of means of each traits over three test years

**Fig. 3:**
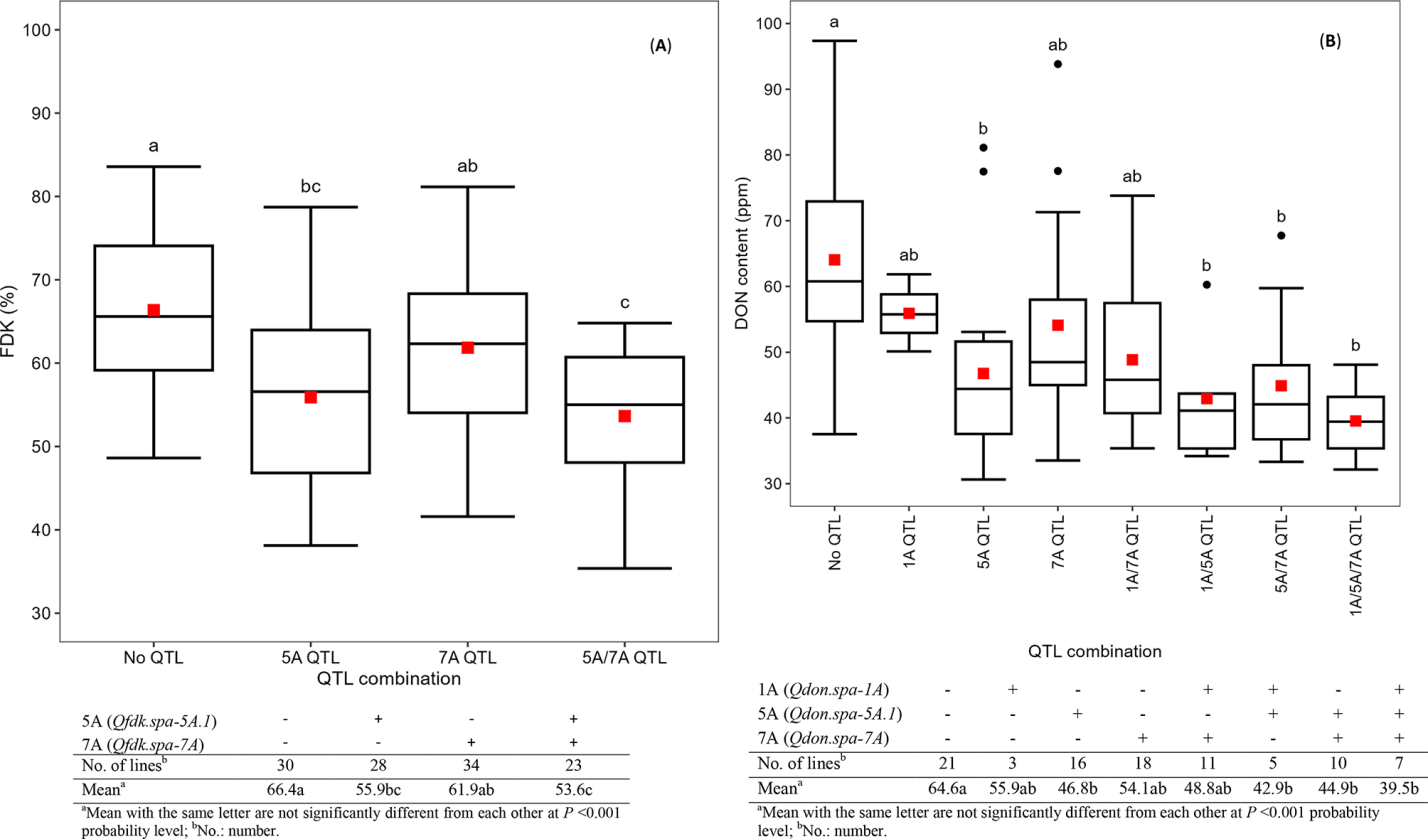
Phenotypic effects of different allele combinations of the 5 and 7 A QTL for FDK (**A**) and of the 1 A, 5 A, and 7 A QTL for DON (**B**). Note: (**A**) 5 A QTL: *Qfdk.spa-5 A.1*; 7 A QTL: *Qfdk.spa-7 A*; (**B**) 1 A QTL: *Qdon.spa-1 A*; 5 A QTL: *Qdon.spa-5 A.1*; 7 A QTL: *Qdon.spa-7 A*. The DON and FDK values used in these comparisons were the averages of the three test years 2015, 2016 and 2017

The lines carrying resistance alleles from either or both *Qfdk.spa-5 A.1* and *Qfdk.spa-7 A* showed significant reduction of FDK compared with lines lacking the resistance alleles as indicated in Fig. 3A. The mean of FDK for lines possessing the *Qfdk.spa-5 A.1* was lower than the mean of FDK for lines carrying the *Qfdk.spa-7 A*, but no significant difference was detected. The combination of both QTL resulted in lower FDK rates which were significantly lower than FDK rates on the lines possessing the *Qfdk.spa-7 A*, but no significant difference was detected with the lines carrying the *Qfdk.spa-5 A*.1. The lines carrying a single QTL or their combinations showed significantly lower DON content than the lines not carrying any QTL (Fig. 3B), but no significant difference in DON content was detected between lines carrying one of the *Qdon.spa-1 A*, *Qdon.spa-5 A.1* or the *Qdon.spa-7 A* QTL. Lines carrying a double QTL combination *Qdon.spa-1 A/ Qdon.spa-5 A.1, Qdon.spa-1 A/ Qdon.spa-7 A* and *Qdon.spa-5 A.1/ Qdon.spa-7 A*, also did not show any significant difference in DON content. The Lines possessing the triple QTL combination *Qdon.spa-1 A/ Qdon.spa-5 A.1/Qdon.spa-7 A* had the lowest DON mean value but only showed a significant reduced DON content compared to the lines possessing no QTL (Fig. 3B).

### KASP marker development

A total of 18 KASP marker designs were generated for each of 5 A QTL (*Qfdk.spa-5 A.1*/*Qdon.spa-5 A.1*) and 7 A QTL (*Qfdk.spa-7 A/Qdon.spa-7 A*) which contribute to both FDK and DON resistance, based on specificity and distribution across the QTL (Table 4). Of the 18 KASP markers associated with the 5 A QTL region (*Qfdk.spa-5 A.1*/*Qdon.spa-5 A.1*), six of the markers are designated as Rank 1 (highest ranking markers based on in silico evaluation), nine markers are Rank 2 and three are Rank 3 (not recommended for use). Of the 18 KASP markers associated with the 7 A QTL region (*Qfdk.spa-7 A/Qdon.spa-7 A*), six of the markers are designated as Rank 1 eleven markers are Rank 2 and one is Rank 3. Results on the technical validation of all 36 KASP markers using a Fluidigm Biomark HD on a subset of the DT707/DT696 DH population are shown in Table 4.

**Table 4:** Breeder-friendly KASP markers developed for two of the most important QTL on chromosomes 5 A (Qfdk/don.spa-5 A.1) and 7 A (Qfdk/don.spa-7 A)

KASP Name	Chr.^a^	Allele Specific 1	Allele Specific 2	Common	Rank	SNP Type	Call Rate (%)	Confidence (%)
DK1872	5 A	atttggaggtcctagagccT	atttggaggtcctagagccC	gagctccgactcgaaggaat	1	Non-homoeologous	93.7	96.4
DK1873	5 A	acgcattttgttcttccattcA	acgcattttgttcttccattcG	aagggagaaaccgatgtccg	1	Non-homoeologous	95.8	99.6
DK1875	5 A	gcaaaccacagaacggagaaaT	gcaaaccacagaacggagaaaG	tccacttgacgtgaagctcg	1	Non-homoeologous	95.8	99.7
DK1879	5 A	gccgacaaagaaacgaaaatcaA	gccgacaaagaaacgaaaatcaT	cgaattcgcgctctacaacg	1	Non-homoeologous	95.8	99.8
DK1884	5 A	cctctttatatgatacgatgatggT	cctctttatatgatacgatgatggC	cggccggaacactgatatga	1	Non-homoeologous	94.7	99.7
DK1885	5 A	actagcacaaatacaacacaccC	actagcacaaatacaacacaccT	gtggttaggtttggtagtcagt	1	Non-homoeologous	69.5	95.9
DK1870	5 A	caacccaatacaacactacaatagT	caacccaatacaacactacaatagC	agtcagaacgatgatccgga	2	Non-homoeologous	94.7	96.8
DK1876	5 A	aacaatcaaaagtacgaactgatgA	aacaatcaaaagtacgaactgatgG	tgctcctgctgttggatatgt	2	Homoeologous	90.5	99.7
DK1877	5 A	gcatctttttggagcaaccG	gcatctttttggagcaaccA	gcggtagatgtaacaccgct	2	Non-homoeologous	94.7	98.5
DK1878	5 A	ttagtttaatatcagcatggtggC	ttagtttaatatcagcatggtggT	tgcatcacacagactagttgga	2	Non-homoeologous	95.8	95.6
DK1880	5 A	agttaagcatgtcatggacttgG	agttaagcatgtcatggacttgA	tggcatgagcatattaagcaaatca	2	Non-homoeologous	95.8	96.6
DK1882	5 A	gcaagcttcttcattgactcacG	gcaagcttcttcattgactcacA	accgctgaatgttgctggat	2	Non-homoeologous	95.8	99.5
DK1883	5 A	atagcaatgaaggcacgcaA	atagcaatgaaggcacgcaG	gccacatgaccatctgcact	2	Non-homoeologous	95.8	96.3
DK1886	5 A	ctctcagccacctacctcaT	ctctcagccacctacctcaC	tgttgactgtcacactgggc	2	Homoeologous	93.7	95.4
DK1887	5 A	tctcttctccggatctcgtG	tctcttctccggatctcgtC	acgatccaaggaagccttcg	2	Non-homoeologous	95.8	99.7
DK1871	5 A	caatgtctcgccaacactcG	caatgtctcgccaacactcA	cacgaaggcatacacggagg	3	Non-homoeologous	0	0
DK1874	5 A	gcttcaataccttggtacttacgG	gcttcaataccttggtacttacgA	cccgaccattaatcttaaggtgt	3	Non-homoeologous	75.8	91.5
DK1881	5 A	tgtcgattcgaaaaactattcactG	tgtcgattcgaaaaactattcactC	actcgcaacgccaagacata	3	Non-homoeologous	88.4	97.9
DK1892	7 A	actaggccttcagttgctctG	actaggccttcagttgctctA	tggcctgcacatctcacag	1	Non-homoeologous	93.7	98.2
DK1894	7 A	acagcagaaagtttacctggacT	acagcagaaagtttacctggacA	gactgcagtacctccacgaa	1	Non-homoeologous	0	0
DK1896	7 A	gcaggaaacgagctatgccA	gcaggaaacgagctatgccG	aattgcggtgacacctccac	1	Non-homoeologous	0	0
DK1897	7 A	cccctactactagatgcgcaC	cccctactactagatgcgcaA	tgtgttctcattgacctccca	1	Non-homoeologous	91.6	96.1
DK1902	7 A	cggtctgaaatgttacccagtT	cggtctgaaatgttacccagtC	cgggccaaatacaacatcgc	1	Non-homoeologous	53.7	90.7
DK1904	7 A	accatctgagttggcaccaG	accatctgagttggcaccaA	tcgacaagttcctcgtacgtg	1	Non-homoeologous	94.7	99.4
DK1888	7 A	gtgtcggtctataggactctactG	gtgtcggtctataggactctactA	cctctgcctaacttggatgca	2	Homoeologous	76	96.2
DK1889	7 A	tcatatgcgacaacttccgT	tcatatgcgacaacttccgG	gggaggaccgtacaacttga	2	Non-homoeologous	0	0
DK1890	7 A	tcatttcagggggatatggagtaT	tcatttcagggggatatggagtaA	acgcccgcttcagtaaaact	2	Non-homoeologous	93.7	98
DK1893	7 A	ccgacgagacacaaagccaA	ccgacgagacacaaagccaG	atcgtgatcatagccagcgg	2	Non-homoeologous	94.7	97.57
DK1895	7 A	gctgctggtccaagtcaagT	gctgctggtccaagtcaagC	cccttctggctcaggttcag	2	Non-homoeologous	65.2	86.7
DK1898	7 A	aaacacccttgtgcaaactcC	aaacacccttgtgcaaactcT	cagctggtaaacacttgggc	2	Homoeologous	92.6	92.8
DK1899	7 A	ggtttgggacgttctttgactG	ggtttgggacgttctttgactA	ggcaaggagacggagaacat	2	Non-homoeologous	95.8	99.5
DK1900	7 A	tgaacagatacatgtaggatggtC	tgaacagatacatgtaggatggtG	ggaactgacgcatcgacctt	2	Non-homoeologous	94.7	98.1
DK1901	7 A	tgagcgcttgtgtctgtactA	tgagcgcttgtgtctgtactG	tggacgtcgaacttggacat	2	Non-homoeologous	0	0
DK1903	7 A	gggagaaaatcgtggtaatttttcG	gggagaaaatcgtggtaatttttcA	accacaacaacctcaccctt	2	Non-homoeologous	63.2	99
DK1905	7 A	agtcctgttggtgtaggtcG	agtcctgttggtgtaggtcC	aagctgaggaatgccatgct	2	Non-homoeologous	0	0
DK1891	7 A	tgttccatatgttaagcatgcttcA	tgttccatatgttaagcatgcttcG	tcgttcttgtccagttaagagat	3	Non-homoeologous	92.6	99.1

^a^Chr.: chromosome

### Physical mapping to the durum wheat reference genome and gene annotation

The physical map position of SNP markers associated with each significant LOD interval in the durum wheat reference genome of Svevo [37] and high-confidence disease related candidate genes in respective regions are presented in Table 5 and Tables S1-S6 of additional file 1. Within the QTL intervals, 81 candidate genes were found that encode proteins with motifs known to be associated with disease resistance such as lignin and cellulose pathway genes, for example, *TRITD5Av1G142630* which is located in the *Qdon.spa-5 A.1* region could be responsible for cellulose synthesis (Table S1 of additional file 1).

**Table 5:** Physical range of each QTL, QTL Confidence Interval (CI) on Svevo physical map, number of high-confidence and low-confidence genes in the QTL interval

QTL	Chr.^a^	Flanking SNP name	SNP Position (cM)	SNP start on Svevo (bp)	SNP end on Svevo (bp)	QTL CI on Svevo physical map(Mbp)	Number of high-confidence genes in QTL region	Number of low-confidence genes in QTL region
*Qdon/fdk.spa-7 A*	7 A	BobWhite_rep_c49367_405	76.03	637,619,108	637,619,208	26.3	218	940
	7 A	wsnp_Ex_c1309_2502521	86.33	663,973,779	663,973,958			
*Qdon.spa-1 A*	1 A	Excalibur_c2389_361	44.21	152,280,937	152,281,037	607.5	2813	14,195
	1 A	BS00062869_51	58.86	759,800,700	759,800,800			
*Qfdk.spa-5 A.1*	5 A	Tdurum_contig5481_369	8.06	395,919,866	395,919,966	22.1	185	688
	5 A	wsnp_Ku_c28245_38183393	19.29	418,018,526	418,018,726			
*Qdon.spa-5 A.1*	5 A	Ra_c28381_461	2.16	109,076,036	109,076,136	302.3	1185	9180
	5 A	BS00067453_51	16.9	411,415,901	411,415,998			
*Qdon.spa-5 A.2*	5 A	BobWhite_c18287_327	2.16	105,927,987	105,928,087	300.2	1164	9122
	5 A	BS00034704_51	14.76	406,199,836	406,199,936			

^a^Chr.: chromosome

## Discussion

Quantitative variation of FDK and DON was evident in the DT707/DT696 DH progeny revealed by continuous distributions. The variations observed among the population lines for both traits across the test years indicates the expression of FDK and DON resistance is influenced by environment. In the early July of each test year, the DT707/DT696 population started the anthesis stage at which wheat is the most susceptible to *Fusarium* infection [16]. Looking at the weather data of three years in July (Table S7 of additional file 1), a temperature of 20.2 to 33.1 °C with a rainfall of 38.3 to 108.9 mm was observed, which indicated these levels of combination of temperature and rainfall could be favorable to FHB development and also cause the differences observed in the FDK and DON values during test years. The enhanced level of resistance by the DT696 across years was in line with the reduced levels of FDK and DON values exhibited by this line compared to DT707 which had relatively high FDK and DON values. This also agrees with the finding of Sari et al. [34] who reported better FHB resistance level of DT696 with lower incidence and severity than DT707 by using the whole set of DT707/DT696 DH population. This indicated that a subset of 120 DH lines from extreme sides served similar purpose to the whole set. As the population studied is a biparental DH population and from extreme sides similar to the case of bulk segregant population, each allele at every locus is likely replicated in about half of the subset of lines at the genetic level, which could increase the reliability of the data analysis on target loci.

Differences in FDK and DON distributions from year to year as indicated by low correlations among years demonstrated a variable response of resistance to different environmental conditions. The inconsistency in QTL detection across years in the current study additionally stresses the effect of environment on the expression of resistant genes and the complexity of the underling mechanisms and the importance of testing in multiple environments. These results agree with previous studies where the authors describe the high dependence of FHB disease expression upon environmental conditions and inconsistencies in QTL expressions in different environments that have frequently been observed in different FHB resistance studies in wheat [11, 38–42].

The highly significant and moderate to strong correlations between FDK and DON observed in three years (Table 2) suggests a common genetic influence and indicates that FDK could be a good predictor of DON content. Several previous studies reported the high correlation between FDK and DON [24]. In a meta-analysis to determine the magnitude, significance, and precision of the association between DON and FHB related traits including incidence, severity and FDK, Paul et al. [43] found that FDK has a strong relationship with DON, with a mean correlation coefficient of 0.73. Similar results were reported by Mwaniki [44] where the correlation between FDK and DON was r = 0.95, although it is hard to make conclusions considering the complex nature of the genetic factors involved in FHB resistance. The correlations are mostly consistent with our QTL mapping results indicating the possibility of colocation of QTL for FDK and DON on chromosomes 5 and 7 A.

Our detection of QTL for FDK and DON is consistent with previous studies. QTL on 5 A for FDK and DON, along with FHB incidence and severity, are reported in a diverse array of wheat germplasm [11, 45]. Our results showing that DT696 contributed alleles for reduced DON content at *Qdon.spa-5 A.1* and *Qdon.spa-5 A.2* is in line with other research findings that report the presence of more than one FHB resistance loci on chromosome 5 A [11, 46, 47], a few of which are associated with FDK and/or DON resistance [48]. Pirseyedi et al. [49] report a QTL for reduced FDK on chromosome 5 A contributed by the durum wheat cultivar Ben. Zhao et al. [50] successfully introgressed the hexaploid *Qfhb.ndwp-5 A* QTL into durum wheat resulting in improved FHB severity and DON content resistance. In a previous study conducted on the DT707/DT696 population using the same SNP map used in our study, DT696 contributes resistance alleles at two locations on chromosome 5 A (5A1 and 5A2) for reduced FHB severity and incidence [34]. Markers flanking QTL *Qdon.spa-5 A.1* and *Qfdk.spa-5 A.1* are located within the interval of the 5A1 FHB resistance QTL contributed by DT696 on chromosome 5 A reported by Sari et al. [34]. This suggests that the same DT696 genetic factors involved in reducing FHB incidence and severity are also involved in reducing FDK and DON.

Comparing the relative position of QTL associated markers on the high density tetraploid consensus map [36], the *Qfdk.spa-5 A.1* and *Qdon.spa-5 A.1* contributed by DT696 most likely are different from those reported by Steiner et al. [51] at *Qfhs.ifa*-*5AS* and *Qfhs.ifa*-*5Ac* from line CM-82,036. The SSR markers flanking *Qfhs.ifa*-*5AS* and *Qfhs.ifa*-*5Ac* [51] and the SNP markers associated with FDK and DON QTL *Qfdk.spa-5 A.1* and *Qdon.spa-5 A.1* QTL identified in the current study do not map within the interval of the QTL identified. Moreover, when we physically mapped the SSR and SNP markers to the Svevo durum wheat reference genome [37] we confirmed that the *Qfdk.spa-5 A.1* and *Qdon.spa-5 A.1* contributed by DT696 are not within the physical range of the FHB QTL contributed by CM-82,036 on chromosome 5 A. Although the novelty of the QTL is unknown, it is very likely that *Qfdk.spa-5 A.1* and *Qdon.spa-5 A.1* are distinct from *Qfhs.ifa*-*5AS* and *Qfhs.ifa*-*5Ac*.

**Fig. 4:**
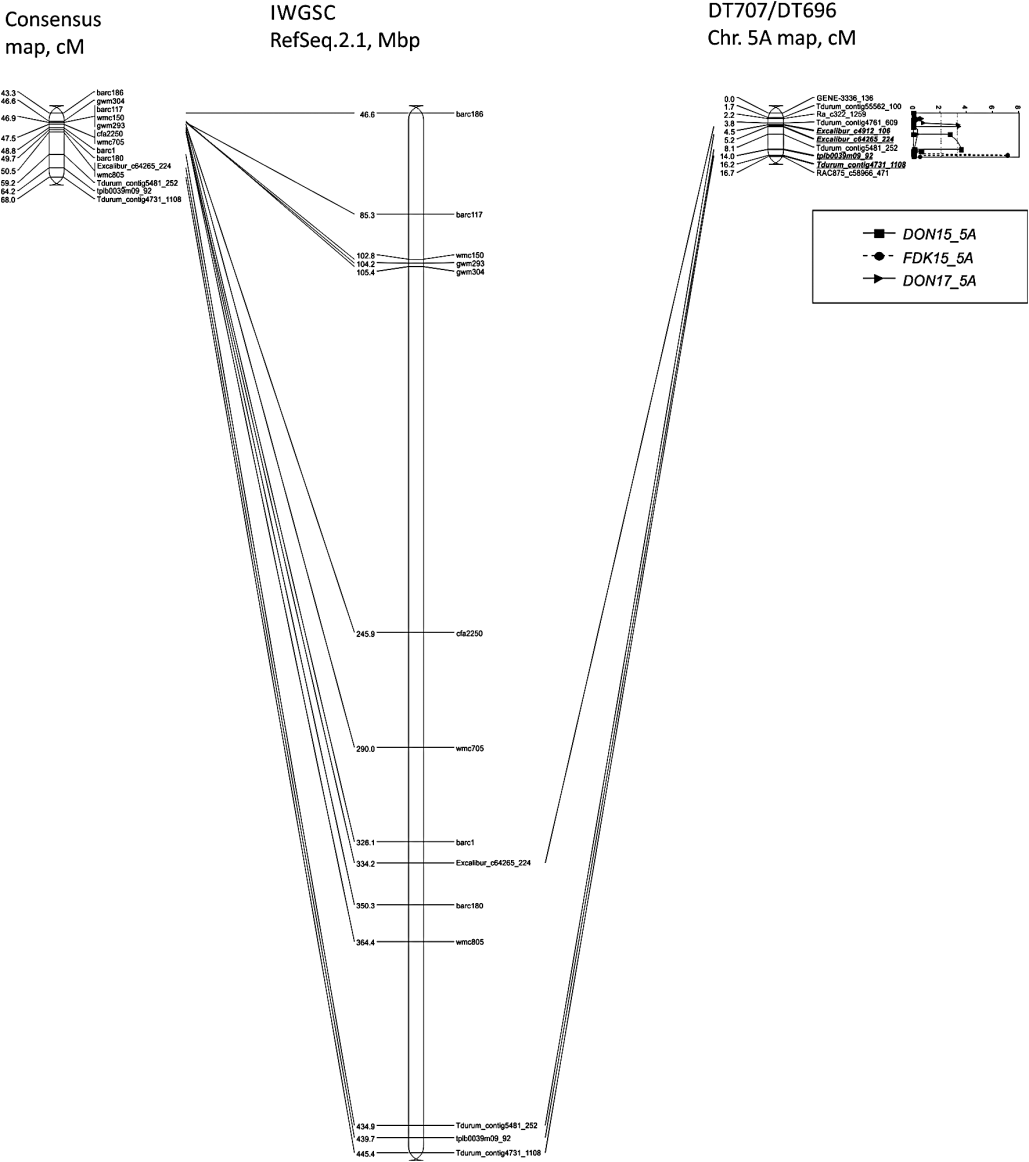
Comparison of markers associated with DON and FDK QTL in high-density tetraploid consensus map (Maccafferri et al., 2014), Chinese Spring IWGSC RefSeq v.2.1 and DT707/DT696 genetic map of wheat chromosome 5A

In 2017, the *Qdon.spa-5 A.2* QTL for lower DON accumulation was mapped on a different region from *Qfdk.spa-5 A.1* and *Qdon.spa-5 A.1*. *Qdon.spa-5 A.1* flanking marker *Tdurum_contig5481_252* was placed at 434.9 Mb and *tplb0039m09_92* at 439.7 in the Chinese Spring wheat genome reference assembly refseq V2.1, whereas *Qdon.spa-5 A.2* associated marker *Excalibur_c64265_224* was placed at 334.2 Mb suggesting two different loci (Fig. 4). In contrary, *Qfdk.spa-5 A.1* marker *Tdurum_contig4731_1108* at 445.4 Mb overlaps with the interval for *Qdon.spa-5 A.1. BS00109052_51* 445.5 Mb and *RAC875_c58966_471* 450.4 Mb, two markers that were associated with FHB incidence and severity in DT696 in the DT707/DT696 population was reported by Sari et al. [34] in a similar interval. The SNP marker at the peak of *Qdon.spa-5 A.2* mapped at 8.8 cM from the SNP marker at the peak of *Qfdk.spa-5 A.1* and *Qdon.spa-5 A.1* which corresponds to a 13.7 cM genetic distance on the high-density tetraploid consensus map and a 73.2 Mb physical distance on the durum wheat reference genome of Svevo [37] (Fig. 4). This result suggests that DT696 is likely contributing alleles for low DON at two different loci on chromosome 5 A. Moreover, this QTL was expressed in a different environment than *Qdon.spa-5 A.1* implying that it likely corresponds to the expression of a different allele. Further study with larger mapping population size will be needed to refine the number of resistance factors in this 5 A region.

The SSR marker *wmc805* in the FHB resistance QTL, *Qfhs.ifa-5 A*, identified by Buerstmayr et al. [52, 53] coincides with the *Qdon.spa-5 A.2* QTL SNP marker *Excalibur_c64265_224* on the high-density tetraploid consensus map [36] (Fig. 4). This finding implies that the QTL for reducing DON *Qdon.spa-5 A.2* from the durum wheat line DT696 overlaps with the QTL for FHB resistance *Qfhs.ifa*-*5 A* from a hexaploid wheat line CM-82,036. Such presence of common genetic basis for FHB component resistance shared between durum wheat and hexaploid wheat have been indicated in different studies [16, 54]. Additionally, many of the QTL identified in tetraploid wheat overlapped with QTL previously detected in hexaploid wheat pointing towards similar genetic control mechanisms of Fusarium resistance in the tetraploid and hexaploid gene pool [48].

At *Qfhs.ifa*-*5 A*, Steiner et al. [51] reported a co-location with anther retention QTL which suggests the colocation of the *Qdon.spa-5 A.2* identified in the current study and the height QTL previously identified by Sari et al. [34] on DT707/DT696 population. Plant height and anther retention affects the likelihood for the fungal spores to enter the florets and modulates conditions for fungal growth rather than inducing active physiological responses in the host, thereby contributing to passive FHB resistance [48].

Despite being environment sensitive, the DT696 QTL on chromosome 7 A, *Qfdk.spa-7 A* and *Qdon.spa-7 A* contributed a substantial amount of phenotypic variation in reducing FDK and DON. In the study undertaken by Sari et al. [34] evaluating the genetic control of FHB visual symptoms, DT696 carried resistance to FHB severity and index on chromosome 7 A. Map comparison revealed that markers *TA006231-0789* and *Kukri_c19696_60* flanking *Qdon.spa-7 A* and *Qfdk.spa-7 A* coincide with the markers associated with FHB resistance QTL identified by Sari et al. [34] and are most likely the same. Moreover, Sari et al. [34] mapped maturity QTL at the same position of the FHB resistance QTL on chromosome 7 A indicating that FHB resistance QTL, low DON and FDK QTL, and late maturity QTL co-locate. From a cross involving an emmer wheat, Ruan et al. [33] identified four QTL on chromosome 7 A in which DT737, a DT696 derived line, contributed three QTL for low FHB incidence, index and visual rating index. Various studies have identified genomic factors responsible for resistance to FHB components on chromosome 7 A and most of them on hexaploid wheat. The 7 A resistance in durum wheat comes from the introgression of hexaploid wheat or exotic gene pools [55]. For example, a study by Kumar et al. [56] reports a putative FHB resistance QTL on chromosome 7 A from a cross between the durum cv. Langdon and the *Triticum dicoccoides* accession PI478742 chromosome 7 A substitution line. Also, Zhao et al. [50] detected QTL for FHB severity resistance on chromosome 7 A contributed by 10Ae564, a durum wheat introgression line with FHB resistance derived from the hexaploid wheat line PI277012.

DT696 contributed alleles for low DON on chromosome 1 A (*Qdon.spa-1 A*). A few previous mapping studies detected FHB resistance QTL on chromosome 1 A [34, 49]. The physical locations of the markers flanking the resistance QTL related to visual symptoms derived from *T. turgidum ssp. carthlicum* cv. Blackbird does not overlap with the physical location of the markers flanking *Qdon.spa-1 A* identified in the present study. A map comparison using the durum wheat reference genome of Svevo between DArT markers associated with reduced DON QTL *Qfhb.ndsu-1 A* from an FHB susceptible durum wheat cultivar Ben reported by Pirseyedi et al. [49] and SNP markers associated with the *Qdon.spa-1 A* from DT696 are at 331.6 bp distant from each other suggesting distinct resistance loci.

In this study QTL *Qfdk.spa-5 A.1*, *Qdon.spa-5 A.1, Qdon.spa-7 A*, and *Qfdk.spa-7 A* were prioritized for the development of SNP-KASP assays (Table 4). The SNP coverage on the 5 and 7 A QTL regions allowed the generation of 36 KASP which will need to be validated using durum germplasm having diverse genetic background to enhance marker assisted breeding for DON and FDK. However, in the validation using the DT707/DT969 population, these KASP markers can effectively select desirable alleles for reducing FDK and DON at the 5 and 7 A QTL to verify their applicability in breeding.

The combined QTL effects for 5 and 7 A in reducing FDK values compared with individual QTL and lines carrying no QTL (Fig. 3A) affirms the QTL are real effect factors. However, the significant LOD values observed in 2015, and the marginal LOD values observed in 2016 and 2017 experiment years with the 5 and 7 A FDK QTL indicates that the trait is highly influenced by changes in the environment. Similar results were also observed with the combined effects of 1 A, 5 and 7 A that resulted in reduced DON concentration compared with the control and individual QTL effects indicating the three QTL were in additive interaction.

Genes underlying resistance to DON and FDK remain not well elucidated and this study investigated the QTL associated with DON and FDK resistance. We observed that the QTL acting alone had a limited and non significant effect on reducing DON and FDK infection, whereas when the QTL were combined the infection levels were reduced significantly. Therefore, the combination of resistance alleles is an effective strategy for enhancing resistance in durum wheat cultivars. Somers et al. [57] found that the 2AL and 5AS QTL on the tetraploid genome had little or no effect in reducing FHB infection when they were alone, but the FHB infection level was much lower if either of them was combined with another FHB resistance QTL on chromosome 6BS. This resistance enhancement could be accomplished by transgressive segregation using an appropriate combination of alleles at different QTL [58, 59]. Transgressive segregation is a key factor for resistant cultivar development. Thus, breeders may be able to develop cultivars with enhanced resistance by use of transgressive segregation of FHB resistance to pyramid different genes through crossing [25, 45, 60, 61].

Multiple potential candidate genes underlying each QTL were identified, such as disease resistance nucleotide binding sites and leucine rich repeats (NBS-LRR), NAC domain and F-box domain containing proteins, WRKY, bZIP, MYB transcription factor, leucine rich repeat receptor kinases (LRR-RK), phytohormone and flavonoid pathway genes and glutathione S-transferase (GST) [3, 62–71]. These candidate genes have been previously reported to be involved in FHB resistance in wheat [62, 63]. Other studies have found ethylene response factor (ERF) and auxin response factors (ARFs) were associated with FHB susceptibility in wheat [64, 65]. A pleiotropic drug resistance (PDR) ABC transporter, TaPDR1, located on chromosome 5 A, was reported to be likely the candidate gene responsible for conferring DON accumulation in hexaploid wheat landrace Wangshuibai [66]. *TRITD1Av1G227130* and *TRITD5Av1G142290*, the genes encoding ERF were identified in the interval of *Qfdon.spa-1 A* and *Qdon.spa-5 A.1*. *TRITD7Av1G245930* in the interval of *Qdon.spa-7 A* encodes an ARF. Multiple genes encoding PDR ABC transporter (*TRITD5Av1G117930*, *TRITD5Av1G117960*, *TRITD5Av1G117970*) were identified in the interval of *Qdon.spa-5 A.2* on 5 A, suggesting that they are the potential candidate genes for resistance to DON accumulation.

Cellulose synthase plays an important role in plant cell wall mediated immunity [67]. TRITD5Av1G142630, encoding a cellulose synthase, could be located in the interval of *Qdon.spa-5 A.1*. Studies have shown that sugar transporters play a key role in the host-pathogen interaction, for example, the *Lr67* gene, encoding a predicted hexose transporter, confers partial resistance to all three wheat rust pathogen species and powdery mildew in wheat [68]. In this study, three genes encoding sugar transporter (*TRITD5Av1G144140*, *TRITD5Av1G144190*, *TRITD5Av1G144280*) reside in the interval of *Qdon.spa-5 A.1*.

Durum wheat has narrower genetic variation for resistance to FHB and FHB associated traits compared to hexaploid wheat. To overcome this narrow genetic variation in durum wheat, many studies have been conducted to introgress resistance from wild or cultivated relatives to durum what, e.g. *T. dicoccoides*, *T. dicoccum, T. elongatum* and *T. carthlicum* [13, 18, 19, 33, 69, 70, 72, 73]. Introgression of exotic genes into elite germplasm might be accompanied with the transfer of undesired morphological trait, thus mining and stacking minor-effect resistance QTL from adapted durum germplasm will contribute to enrich the gene pool and improve resistance levels without compromising the elite germplasm characteristics.

## Conclusion

In conclusion, we identified a QTL on chromosomes 5 and 7 A associated with low FDK and four QTL on 1 A, 5 A (two loci) and 7 A associated with low DON accumulation in the DT707/DT969 durum wheat population. The desirable alleles for the resistance at all the loci derived from DT696, but no significant QTL was detected from the other parent, DT707. The QTL on chromosomes 5 A (*Qfdk.spa-5 A.1, Qdon.spa-5 A.1*) and 7 A (*Qfdk.spa-7 A, Qdon.spa-7 A*) were simultaneously responsible for low FDK and low DON accumulation, whereas the other QTL, 1 A (*Qdon.spa-1 A*) and 5 A (*Qdon.spa-5 A.2*), were only associated with low DON accumulation. The DH lines in which the desired resistance genes occur are useful resources in breeding for FDK and DON resistance in durum wheat. Markers flanking each QTL were physically mapped against the durum wheat reference sequence and candidate genes involved in FDK and DON resistance were identified within the QTL intervals. Breeder-friendly KASP markers were developed for two of the QTL on chromosomes 5 and 7 A and anticipated to be useful markers for selecting for low FDK and DON QTL in durum wheat breeding programs. These KASP markers were effective in selecting resistance alleles of FDK and DON at the 5 and 7 A QTL in using the DT707/DT969 DH population to validate the applicability of markers. The DH lines carrying resistance alleles of the 5 and 7 A QTL showed significant reductions of FDK and DON content compared with lines lacking the resistance alleles based on the average values of three years.

## Methods

### Plant materials

The DT707/DT696 DH population described by Sari et al. [34] of 423 lines was further evaluated in this study. The population was developed from the cross of DT707 and DT696 made in 2001 at the Swift Current Research and Development Centre (SCRDC) of Agriculture and Agri-Food Canada. Line DT707 (also known as 9468-DQ*2) was developed at SCRDC and is derived from a two-way cross AC Avonlea/DT 665. DT665 was derived from a cross between Kyle/Nile. Line DT696 (also known as 9366 BS*1) was developed at the Swift Current Research and Development Centre (SCRDC) of Agriculture and Agri-Food Canada (AAFC) and derived from a three-way cross DT618/DT637//Kyle [21]. The population was produced through a doubled haploid (DH) technique at SCRDC using the maize pollen method described by Humphreys and Knox [74].

### Field trials

The 423 DH lines of the DT707/DT696, the parental lines, and checks were grown at an Agriculture Agri-Food Canada (AAFC) FHB inoculated nursery near Morden (MDN), Manitoba in 2015, 2016 and 2017 and Indian Head, Saskatchewan in 2015. At both Morden and Indian Head, the experiments were conducted as an augmented randomized block design with 19 entries per incomplete block including six FHB standard checks used in FHB evaluation of wheat breeding lines for the wheat variety registration (from resistant to susceptible) and two parents. Sixty seeds were planted per line on 1 m row plots using a six-row cassette Wintersteiger planter as recently described in detail by Berraies et al. [75]. The FHB standard checks AAC Tenacious, 5602 h, FHB37, CDC Teal, AC Morse, and AC Cora were replicated nine times in the experiment. Whereas the parental lines were replicated five times, the DH lines were not replicated. The protocol for the *F. graminearum* corn kernel inoculum preparation was adopted from Gilbert and Woods [76]. The *F. graminearum* isolates used, the method of the inoculum application and the details on data collection were described previously by Berraies et al. [75]. Briefly, the FHB corn inoculum was applied when the plants reached 4–6 leaf stage and repeated a week after the first application, which was followed by third application after 7 days. All the inoculum applications were followed by irrigation to promote disease development. FHB incidence and severity were rated using a 0-100% scale at 21 day post inoculation. The weather condition of the Morden location during the experiment period is summarized in Table S7 of additional file 1.

FHB point inoculation was conducted in the greenhouse at Swift Current Research and Development Centre in 2016 as previously described by Ruan et al. [33]. Briefly five spikes from individual plants of the DT707/DT696 DH population and two parents were inoculated with F. graminearum isolates in three replications. The inoculation was made on a mainstem spike on each plant with 10 µL of macroconidial suspension (50,000 spores/mL) containing 0.02% Tween 20 when the plants reached at 50% anthesis. Disease severity was rated as the percentage of infected spikelets per spike 21 days after inoculation.

Because of the high cost of DON chemical analysis, we selected for DON accumulation analysis, a subset of 120 DH lines from the large population of 423 lines representing the most field resistant and susceptible lines based on severity reaction during 2015. This group of lines was used through the following testing seasons. At maturity the selected 120 lines and three replications of each parent and checks were manually harvested and 25 random heads were threshed with a belt thresher set at low wind speed and hand cleaned to retain all the fusarium infected kernels. FDK was estimated by visually separating and counting chalky and shriveled kernels from the sound kernels from a 10 g random sample of grain from the 25 threshed heads. The percentage of FDK was calculated with the formula: Number of infected kernels/Total number of kernels in 10 g x 100.

For DON quantification, the total grain sample from the twenty-five heads was finely ground with a UDY Cyclone Sample Mill and a 1 g sub-sample was used to quantify DON concentration. To prepare extracts, 5 mL of methanol: water (1:9, vol/vol) was added to the 1 g ground samples in 10-mL plastic tubes. The tubes were then subjected to end-over-end mixing for one hour, then centrifuged for 5 min at 2000 rpm. DON analysis was conducted on the filtrate using the AAFC “in-house” enzyme-linked immunosorbent assay (ELISA) as described by Sinha et al. [77]. The accuracy of the ELISA procedures has been reported to be comparable to that of a gas chromatography method [78]. The limit of quantitation was 0.1 mg kg^− 1^.

### Genotyping and map construction

The 90 K iSelect array developed by Illumina CSPro (San Diego, CA, USA) as described by Wang et al. [35] was used to genotype the two parental lines and the 423 DH lines. Details on the genotyping and high density linkage map construction on this population were reported by Sari et al. (2018). Briefly, the genetic linkage map developed by Sari et al. [34] and used here consisted of 2,943 SNP markers in 19 linkage groups with an average marker density of 0.6 cM. The total length of the map was 1,808.4 cM.

### QTL analysis

The QTL analysis was performed using the MapQTL6® [79] software for the DON and FDK as described in a previous study [75]. In short, the analysis was first conducted using Kruskal–Wallis (KW) test option in the MapQTL to determine the association between marker and FHB traits. This test was followed by a simple interval mapping and selection of automatic cofactor markers. QTL identified to be significant in simple interval mapping were subjected to the multiple QTL mapping (MQM) analysis [80] and the QTL were declared significant at *P* < 0.05.

### Significance thresholds and confidence intervals

Through MapQTL6® we generated genome-wide and chromosome-wide significance thresholds from permutation estimates by dividing the nominal p-value by the total number of linkage groups analyzed in this study. QTL confidence intervals were estimated by 1.3-LOD support interval with 99% confidence interval probability coverage. The thresholds of logarithm of odds (LOD) scores for significant QTL (*P* < 0.05) were determined by performing 1000 permutations on the genome-wide level [75]. Significant QTL and regions were visualized using the software MapChart 2.1.

### SNP markers identification and KASP markers design

To facilitate marker-assisted selection (MAS), SNP markers associated with major QTL for DON and FDK were converted to KASP markers using SNPs identified between DT696 and DT707 and the wheat reduced exome capture sequence (SeqCap EZ Design 160318_Wheat_Tae_Red_EZ_HX1 Roche, Nimblegen) at the National Research Council (NRC), Saskatoon. Genomic libraries were prepared from 1 µg of DNA using “Library Preparation Kit Illumina” (Kapa Biosciences) pooled on an equimolar basis and captured according to the manufacturer’s instructions (Roche Nimblegen). Libraries were sequenced using an Illumina HiSeq 2500 using High Output version 4 chemistry (2 × 125 bp). Reads were trimmed using Trimmomatic v0.32 [81] with the following parameters: phred33 ILLUMINACLIP: all_illumina.fasta:2:20:10:1 TRAILING:20 SLIDINGWINDOW:5:20 MINLEN:70. The trimmed reads were aligned against the IWGSC wheat RefSeq v1.0 using BWA-MEM v0.7.15 with the –M. Both split reads and non-unique mapping reads were removed on the basis of the difference in scoring of primary to secondary alignments. Duplicates were removed with Picard Tools 2.4.1. Variants were called using SAMtools v1.7 [82] mpileup and BCFtools v1.6 [83], and filtered for contrasting homozygous calls, based on an average depth of three reads, MAPQ > 30, QUAL > 20 and variants detected in a Chinese Spring negative control. Using the rough physical interval defined by 90 K markers (5 A: 430–450 Mb, 7 A: 650–670 Mb), all qualifying SNPs (1590 SNPs for 5 A, 1792 SNPs for 7 A) were subjected to KASP assay design using the PolyMarker primer design pipeline [84]. A subset of 18 successful KASP marker designs were selected for each QTL based on predicted specificity (based on an independent BLAST search) near the QTL. The markers were then validated using a Fluidigm Biomark HD with a modified protocol to accommodate KASP chemistry.

### Physical mapping to the durum wheat reference genome and gene annotation

The sequences around the 90 K SNPs were downloaded from the Kansas State University SNP marker database (http://wheatgenomics.plantpath.ksu.edu/snp.html). A BLAST search of the Durum Wheat Genome Database at https://wheat.pw.usda.gov/GG3/node/759 was used to align the sequences of the 90 K SNP markers that localized within each QTL LOD plot interval to the genome sequence of the durum wheat cultivar “Svevo” [37] and identify putative physical intervals. The annotated genes within ± 5 Mb flanking each QTL peak marker were retrieved using the Durum Wheat Genome Browser at https://wheat.pw.usda.gov/GG3/node/759.

### Statistical analysis

Analysis of variance (ANOVA) was performed on FHB traits using a mixed model approach in the Statistical Analysis System (SAS) software version 9.3 [85]. During the analysis, the DH lines were considered the fixed variable, whereas test years were considered the random variable. Spearman’s correlations of FDK and DON within and among years and with FHB incidence and severity were calculated using the PROC CORR function of SAS. The frequency distributions of FDK and DON data of the 120 lines were evaluated for normality. The combined phenotypic effects of QTL associated with three-year averages of FDK and DON content were compared by classifying the lines using markers associated with respective QTL and presented in box plots. Data analysis comparing phenotypic data of the lines and broad-sense heritability values of the traits was performed using R statistical package. Broad-sense heritability was calculated according to Holland et al. [86]. The DH lines were grouped into different classes based on QTL they carry and statistical analysis was conducted to see the effect of QTL combinations in reducing percent FDK and DON content.

### Electronic supplementary material

Below is the link to the electronic supplementary material.

**Additional file 1: Table S1:** Summary of potential candidate genes with high confidence within Fusarium damaged kernels (FDK) and deoxynivalenol (DON) resistance QTL intervals in DT696. **Table S2:** List of annotated genes within Qfdon.spa-1A interval. **Table S3:** List of annotated genes within Qfdk.spa-5A.1 interval. **Table S4:** List of annotated genes within Qdon.spa-5A.1 interval. **Table S5:** List of annotated genes within Qdon.spa-5A.2 interval. **Table S6:** List of annotated genes within Qdon.spa-7A and Qfdk.spa-7A interval. **Table S7:** Weather data for three critical crop growth months at the Morden, Manitoba where the Fusarium head blight field evaluation was conducted from 2015 to 2017. **Table S8:** Spearman?s correlation coefficients between FHB incidence (INC), FHB severity (SEV), Fusarium damaged kernels (FDK) and deoxynivalenol (DON) measured over years and locations on the 120 lines of the DT696/DT707

## Data Availability

The datasets generated or analysed during this study are included in this article and its Additional file or are available from the corresponding author on reasonable request.

## Abbreviations

FHB: Fusarium head blight
FDK: Fusarium damaged kernels
DON: Deoxynivalenol
SNP: Single Nucleotide Polymorphism
QTL: Quantitative Trait Loci
KASP: Kompetitive allele specific PCR
CWAD: Canada Western Amber Durum
